# Starting postgraduate medical training in general practice with a rotation in general practice – a qualitative study on experiences and effects

**DOI:** 10.3205/zma001708

**Published:** 2024-11-15

**Authors:** Christine Becker, Sandra Stengel, Marco Roos, Attila Altiner, Simon Schwill

**Affiliations:** 1 University Hospital Heidelberg, Department of General Practice and Health Services Research, Heidelberg, Germany; 2 University of Augsburg, Faculty of Medicine, Department of General Practice, Augsburg, Germany

**Keywords:** training, general medicine, professional identity formation, self-directed learning

## Abstract

**Objective:**

In Germany, the rotation into the general practitioner’s practice (GPP) as part of postgraduate medical training in general practice traditionally takes place at the end of the training period. The aim of this study was to explore possible subsequent effects of beginning training in the GPP from the perspective of general practitioners (GPs) and GP trainees.

**Methods:**

Nationwide, GPs and GP trainees were recruited who started specialization in GP in the GPP. Semi-structured telephone interviews were conducted between June and October 2022 using a self-developed interview-guide. The results were transcribed verbatim and analyzed using content analysis.

**Results:**

N=15 interviews were conducted, averaging 54 minutes (32-75 minutes) each (9 federal states, 4 GPs, 11 GP trainees). From the participants’ perspective, advantages included close supervision, flat hierarchies, more time for preparation and follow-up, self-directed learning as well as higher basic salary and regular working hours. Positive effects mentioned were reinforcement in career choice, early understanding of workflow in GPP, early development of a GP attitude and strengthening of professional self-confidence. Disadvantages included initial uncertainty at the beginning of the profession and limited opportunities for peer exchange. In conclusion, all participants would recommend beginning specialization with a rotation in the GPP.

**Discussion:**

Starting in GGP allows GP trainees to learn about GP in a self-directed, research-oriented manner and based on consultations which enables early professional identity formation. GP trainees should avoid lack of collegial support by participating in a postgraduate training program. In a second step, GP trainers perspectives need to be assessed.

**Conclusion:**

Beginning GP postgraduate training with a rotation in GP is advantageous and should be structurally promoted.

## Zusammenfassung

**Zielsetzung:**

In der Weiterbildung Allgemeinmedizin in Deutschland wird die Rotation in die hausärztliche Praxis (HÄP) traditionell an das Ende der Weiterbildungszeit gelegt. Ziel dieser Studie war, die Folgeeffekte durch einen Weiterbildungsbeginn in der HÄP aus Sicht von Fachärzt*innen und (FÄ AM) Ärzt*innen in Weiterbildung (ÄiW) zu explorieren.

**Methodik:**

Deutschlandweit wurden FÄ AM und ÄiW rekrutiert, die ihre Weiterbildung in der HÄP begonnen hatten. Zwischen Juni und Oktober 2022 wurden semistrukturierte Telefon-Interviews anhand eines selbst entwickelten Leitfadens durchgeführt. Die Ergebnisse wurden Wort-für-Wort transkribiert und inhaltsanalytisch nach der Methode von Kuckartz ausgewertet.

**Ergebnisse:**

Es wurden N=15 Interviews mit durchschnittlich 54 Min. (32-75 Min.) durchgeführt (9 Bundesländer, 4 FÄ AM, 11 ÄiW AM). Aus Sicht der Teilnehmenden (TN) wurden als Vorteile insbesondere eine enge Supervision, flache Hierarchien und mehr Zeit für Vor- und Nachbereitung, selbstgesteuertes Lernen sowie ein höheres Grundgehalt und regelmäßige Arbeitszeiten genannt. Als positive Effekte wurden eine Bestärkung in der Berufswahl, ein frühes Verständnis der allgemeinmedizinischen Arbeitsweise, die frühe Entwicklung einer hausärztlichen Haltung sowie eine Stärkung des professionellen Selbstbewusstseins genannt. Nachteilig wurden eine zu Berufsbeginn bestehende Unsicherheit und geringe Möglichkeiten des peer-Austausches beschrieben. Im Fazit würden alle TN empfehlen die Weiterbildung in der HÄP zu beginnen.

**Diskussion:**

Der Beginn der Weiterbildung in der HÄP ermöglicht ÄiW selbstgesteuert, forschend und anhand von Beratungsanlässen die allgemeinmedizinische Arbeitsweise kennen zu lernen, was zu einem frühen Zeitpunkt eine professionelle Identitätsbildung in der Allgemeinmedizin ermöglicht. ÄiW sollten *peer*-Austausch in den Kompetenzzentren Weiterbildung erhalten. Die Sicht der Weiterbildungsbefugten sollte ebenfalls erhoben werden.

**Schlussfolgerung:**

Der Beginn der Weiterbildung in der HÄP ist vorteilhaft und sollte strukturell gefördert werden.

## Introduction

In Germany, obtaining specialist certification is individually regulated by the education guidelines for physicians (“Weiterbildungsordnung”, WBO) of the regional medical associations, based on the model education guidelines of the German Medical Association from 2020 [1], [2]. For the specialization in general practice (GP), the current WBO in Baden-Württemberg (BW) requires a training period of 60 months, of which 24 months must be completed in the general practitioner’s practice (GPPP), 12 months in acute inpatient care in internal medicine, 6 months in a field of direct patient care, and a total of 18 months in any areas of direct patient care [3]. GP trainees can freely choose the sequence of rotations.

General practice training (GPT) in Germany has long been considered unattractive, mainly due to lack of support and structure [4], [5]. This has fundamentally changed in recent years with the positive developments in BW through the “Verbundweiterbildung*^Plus^*^®^” program [6], [7]. Since 2017, competence centers (CCs) in general medicine have been established nationwide, offering seminars, mentoring, and peer support for GP trainees in postgraduate training [8]. The structured seminar curriculum offered by the Competence Center for postgraduate training in Baden-Württemberg (“KWBW”) provides guidance for GP trainees [9]. The seminar program was developed in consideration of the competency-based curriculum of the German Society of General and Family Medicine [10].

Beginning GPT in the GPP is uncommon in Germany. It is known that many GP trainees decide to specialize in GP in the course of their training in the third or fourth year of training [11]. Sociodemographic studies in BW have shown that approximately 20% of GP trainees enter GPT directly [12]. Over the years, the financial support for ambulatory training periods by the Association of Statutory Health Insurance Physicians has been expanded in BW [13]. Internationally, beginning GPT in the GPP as the first rotation is common practice, for example in Denmark or the Netherlands [14], [15], [16], [17].

It is unclear how GP trainees and their GP trainers perceive the direct beginning of GPT in the GPP. Studies on this topic are not available in Germany. The aim of this study was to explore the advantages and disadvantages, as well as the subsequent effects of beginning training in the GPP on training and later professional life from the perspective of GP trainers and GP trainees. 

## Methods

### Ethics

The study was approved by the Ethics Committee of Heidelberg University as part of an evaluation of postgraduate medical training in general medicine (Approval No. S-570/2015). Participants provided written consent to participate in the study and to use the interview data in written and pseudonymized form.

### Study design

An exploratory qualitative study was conducted with physicians who were interviewed using semi-structured interviews about their entry into GP. A COREQ checklist, as a reporting guideline, is attached (see attachment 1 ).

### Recruitment

The aim of recruitment was to identify GP trainees who had started their training in GPP. Since beginning training in the GPP is uncommon and only occasionally approved, only a few graduates could be found. Recruitment was expanded to include GP trainees who had completed their first rotation in primary care. Participants were reached through purposive sampling via e-mails throughout Germany. GP trainees were in various stages of training (years 1 to 5 out of 5). The authors were supported in recruitment by the German Society of General and Family Medicine (DEGAM), the Association of Young General Practitioners Germany (JADE) as well as CC in Baden-Württemberg and Bavaria. Via those recruitment strategies a sufficient amount of interview partners could be recruited from multiple federal states. No remuneration was provided to participants. At the time of the interviews, there were no professional relationships between the participants and the authors.

### Development of interview guideline 

The interview guide was developed by three scientists, all experienced in GP and with expertise in medical education (C.B., S.S., S.SC.) (see attachment 2 ). The questions were formulated openly to allow sufficient space for the interviewees to share their own experiences. The guiding questions focused on the experience of entering training in the GPP. Specifically, the effects on further medical education, professional life, development of a professional identity and personal attitudes were of interest. Additionally, the advantages and disadvantages of beginning a career in GP were explored. Another focus was on defining challenges and strategies for encountering and coping with them. The guideline was piloted three times and the clarity of the questions was adjusted accordingly. Prior to the interviews, socio-demographic data were collected.

### Interview procedure

The interviews were conducted between June and October 2022 as semi-structured telephone interviews, using a recording device, by a researcher (C.B.) without the presence of third parties. The researcher introduced herself, explained her role, and presented the research questions both at the workplace and in the researcher's home environment. Subsequently, the recordings were transcribed verbatim by research assistants and irreversibly deleted upon completion of the study. The transcripts were not provided to the participants for correction.

### Data analysis

Data analysis was conducted on paper by two researchers (GPs, male/female, with varying levels of experience in qualitative data analysis) using a content-structuring qualitative content analysis approach following Kuckartz’s method [18]. Category formation was deductive-inductive, with main categories predetermined and subcategories and codes supplemented during the interview analysis.

## Results

### Interview procedure

A total of n=16 interviews were conducted, with an average duration of 54 minutes (min. 32 minutes to max. 75 minutes). One interviewee was excluded as their medical training did not begin exclusively in GP. Thus, n=15 interviews were included in the analysis. No further recruitment was necessary due to the comprehensive data collected.

### Socio-demographic data

Among the participants, n=4 were board-certified GPs. At the time of the interviews, among n=11 GP trainees n=1 was in the first year, n=2 in the second year, n=5 in the third year, n=2 in the fourth year, and n=1 in the fifth year of training (full-time equivalents). All interviewed GP trainees began their training under the current education guidelines for physicians of 2020. Socio-demographic data of the participants are summarized in table 1 (Tab. 1).

### Advantages and disadvantages

Participants reported both advantages and disadvantages, as well as various effects, of starting their careers in primary care. The advantages outweighed the disadvantages significantly. The advantages and disadvantages mentioned by the interviewed physicians are presented in table 2 (Tab. 2) and table 3 (Tab. 3), and their key points are discussed below.

#### Advantages

The pleasant side effect of regular working hours with advantages for individual needs such as reconciliation of work and family life, completing dissertations and a slightly higher basic salary in practice during the first year of residency compared to working in a hospital was highlighted as advantageous. However, significantly more valued advantages were identified, such as the ability to switch perspectives between inpatient and outpatient care in subsequent rotations. This had implications for patient management in clinical rotations. Observing the approach and mediating the attitude of mentors was considered important and influential. The flat hierarchies and communication on an equal footing with mentors, creating a relaxed environment, were described as advantageous for a successful career start.

#### Disadvantages

A notable disadvantage was the professional uncertainty at the beginning of the career, although some participants regarded this as normal when starting a new job. The lack of peer exchange, being often the only trainee in a small practice team, was also perceived as a disadvantage. Respondents recommended younger trainees to connect with regional competence centers and JADE groups to prevent this professional isolation.

### Effects of beginning training in general practice

Further effects of starting in primary care are summarized below in addition to table 4 (Tab. 4) and attachment 3 . 

#### Motivation for the profession and training progression

The experience of beginning training in GPP was perceived as motivating, as it confirmed GP trainees’ choice of specialty early in their careers. Additionally, important competencies required for future practice and individual strengths and weaknesses were identified, influencing further training planning. Participants described a decrease in apprehension about future self-employment. 

#### Understanding the specifics of general practice

Participants described early understanding of GP principles as helpful for future practice in GP and consecutive clinical rotations. The openness, curiosity, and willingness to learn from the model (GP trainers) were seen as advantageous for internalizing these principles. Recognizing the role of GP, its strengths and challenges and understanding interfaces in the healthcare system were considered beneficial for professional development.

#### Development of a professional identity

Participants emphasized the development of a GP identity, personal attitudes, and reflection on their role in the healthcare system, shaped by experiences in GP with their mentors. They valued these experiences for their ongoing training. Additionally, they discovered personal areas of interest, which positively influenced their motivation for professional practice, for example pursuing additional qualifications.

#### Opportunity for self-directed learning

Self-directed and exploratory learning opportunities were described as promoters for an enhanced earning success. Participants appreciated the ability to control and increase the complexity of patient encounters and the supportive environment in GP, such as time for self-study or easy access to mentors for questions. Satisfaction and personal reinforcement were associated with assuming responsibility and developing problem-solving skills through increasingly autonomous work.

#### Positive impact on self-confidence

Experiences during this initial phase of professional practice led to increased self-confidence through the realization of one’s efficacy. Recognizing one's own value and identifying specific learning goals contributed to greater self-assurance in subsequent rotations, such as advocating for quality training aligned with the needs of GP or negotiating employment contracts.

## Discussion

This study successfully explored the research questions, highlighting several advantageous aspects of beginning GP training in the GPP. Notably, participants found confirmation of their choice of specialty, clarity regarding learning objectives in GP and an early understanding and adoption of GP working approaches as particularly beneficial and motivating. Additionally, participants emphasized the early development of a professional identity, reflective practices regarding their role in the healthcare system and the discovery of personal interests, contributing to job satisfaction. The opportunity for self-directed and exploratory learning was also highlighted as advantageous. Participants reported a strengthening of self-confidence and a significant sense of self-efficacy resulting from their experiences.

However, disadvantages such as professional uncertainty at the start of the career and the lack of peer exchange were noted. Nevertheless, all participants recommended beginning GP training in a GPP. The reported effects present an opportunity to recruit GP trainees early in their careers. It is noteworthy that eleven participants had already acquired experience in general practice during their final year of study as part of their clinical rotations (“Praktisches Jahr”). This underscores the importance of promoting a positive perception of GP during basic medical education [19], [20], [21]. By providing attractive teaching opportunities at universities and expanding practical training opportunities, there is potential to attract more young physicians directly into GP.

Early exposure to the peculiarities of GP work seems to positively influence further learning. Although these principles and identity development can occur later in training, the early integration of experiences in GP may offer a promising option for structuring postgraduate training [22]. The interviewed physicians unanimously emphasized the importance of subsequent rotations in other specialties to acquire complementary competencies and enhance communication between specialists to improve patient care [23].

Peer exchange was considered highly important, both during training in the GPP, where trainees often lack an immediate peer group, and during clinical rotations, where contact with general practice might otherwise be limited. This appears to be crucial for the development of a GP identity and is facilitated in other countries, such as Denmark, through weekly work in primary care practices during hospital rotations [14], [24]. 

A notable effect reported by some participants was the strengthening of professional self-confidence through their initial GP rotation. This sense of self-efficacy, coupled with the realization of possessing competencies early in their careers, seemed to positively influence trainees' assertiveness in subsequent rotations. The shaping of a GP identity and learning through role models, particularly within GPP under the guidance of a GP trainer, appears beneficial in the development of professional identity [25]. An early initiation of this process is especially feasible within primary care practices.

Concerning disadvantages, participants noted professional uncertainty at the beginning of their careers and the associated limited decision-making capacity. However, this was regarded as a normal aspect of starting a new job, and it was suggested that good supervision and self-directed learning could effectively address this challenge. Participants also reported that those transitioning from hospital settings sometimes encountered challenges with the GP approach, requiring a period of adjustment due to learned working methods. Another issue, in addition to the previously mentioned lack of peer exchange, was the potential lack of practical exposure before the specialty exam. This problem could be addressed by splitting the 24 months rotation in GPP between the beginning and end of the program.

For mentors the benefits of having a trainee included the perceived relief of workload and the opportunity for mutual learning. Although increased supervision may initially pose challenges, the enthusiasm and inquisitiveness of young residents were viewed as opportunities for self-reflection and knowledge exchange.

In conclusion, starting residency training in GP offers various advantages, including motivational benefits, identity development and opportunities for self-directed learning. Addressing challenges such as professional uncertainty and limited peer exchange requires thoughtful program design and mentorship. Overall, integrating GP experiences early in postgraduate training can contribute to the recruitment and retention of future general practitioners.

For mentors, competencies centers for postgraduate training offer train-the-trainer seminars [26] that can enable peer-exchange for GP trainers as well as mediate possible ways to deal with possible problems opposed by GP trainees starting training in the GPP. 

### Strengths

To date, there is no comparable study in Germany examining the effects of beginning postgraduate training in GP in the GPP, whereas this practice has been established abroad for some time. Noteworthy to the authors is the overall homogeneous responses from colleagues regarding the affirmation of their career choice and the importance of embedding the mindset related to the development of a professional identity. Additionally, the emphasis on the influence on the progression of training appears as an opportunity to strengthen the field of general practice and potentially contribute to long-term healthcare provision.

### Limitations

Some effects observed in this study may also occur later in the course of postgraduate training, such as the development of a professional attitude. However, the potential for influence and guidance at this late stage is questionable. This qualitative study surveyed a sample of GP trainees and young GPs, the majority of whom had already completed their clinical rotations in GP during their final year of medical school. A comparison group could not be examined within the scope of this qualitative study, which could be a subject of further research. Additionally, the effects, advantages, and disadvantages were not explored from the perspective of the supervising GP trainers.

## Conclusion

These findings can encourage attending physicians to hire young GP trainees in their first year of training and seize the opportunity to significantly influence the next generation of general practitioners. Those young trainees who aim for direct entry into GP appear highly motivated and eager to learn. Similarly, consideration should be given to expanding clinical rotation opportunities in GP during the final year of medical school to inspire students to pursue careers in the field. Given the associated demands and potential challenges for GP trainers, their perspectives regarding the reasons for and against hiring trainees in their first year of training should be explored in the next step.

## Authors’ ORCIDs

- Christine Becker: [0009-0009-4802-5761]
- Sandra Stengel: [0000-0002-4400-7605]
- Marco Roos: [0000-0003-1596-5908]
- Attila Altiner: [0000-0002-2429-933X]
- Simon Schwill: [0000-0002-0954-2194]

## Acknowledgements

We would like to express our gratitude to the DEGAM Section for postgraduate training, the CC for postgraduate training in Bavaria (Kompetenzzentrum Weiterbildung Allgemeinmedizin Bayern, KWAB) and Baden-Württemberg (KWBW Verbundweiterbildungplus), as well as the volunteer colleagues of JADE, for their assistance in recruitment. We acknowledge the support of Dr. Benedikt Sonnek for English translation of this manuscript.

## Competing interests

The authors declare that they have no competing interests. 

## Supplementary Material

COREQ (Consolidated criteria for Reporting Qualitive research) Checklist

Interview guide for general practitioners who have started their training in a general practitioners office

Effects of starting postgraduate training at a general practitioner’s practice – key quotes

## Figures and Tables

**Table 1:**
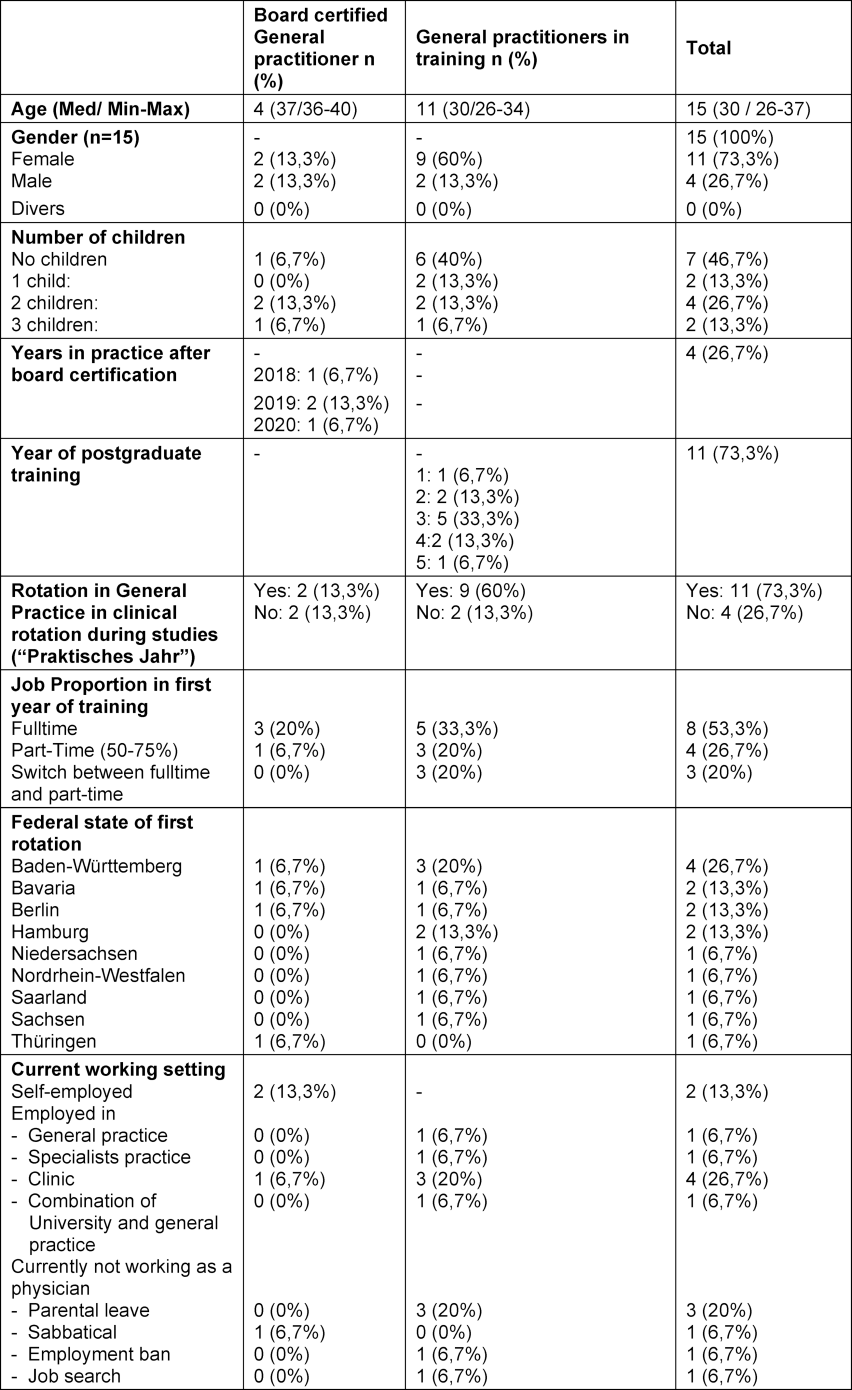
Socio-demographic data of physicians beginning postgraduate medical training in the general practitioner’s practice (n=15)

**Table 2:**
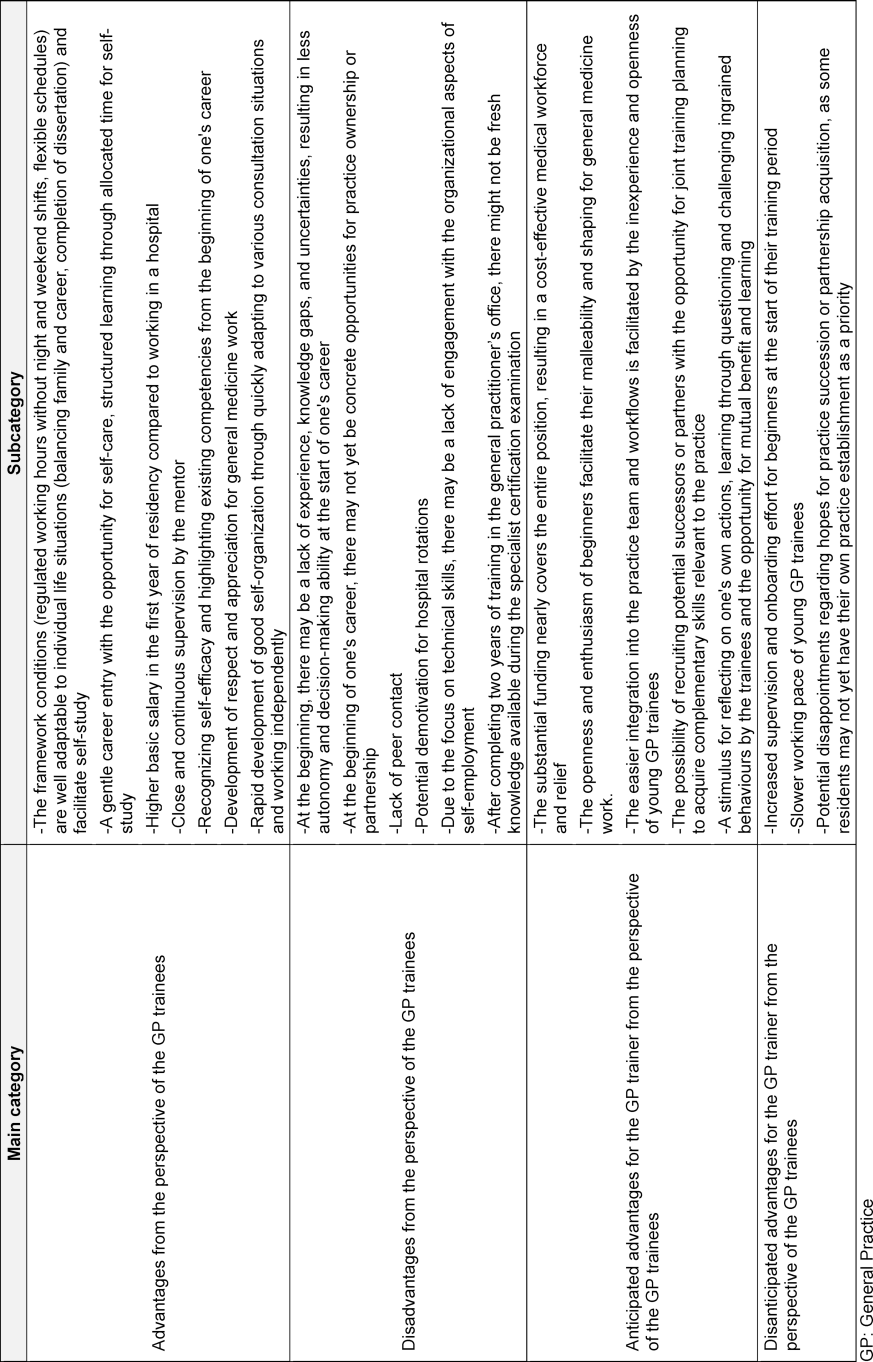
Advantages and disadvantages of starting specialisation in general practice in a general practitioner’s practice from the perspective of GP trainees

**Table 3:**
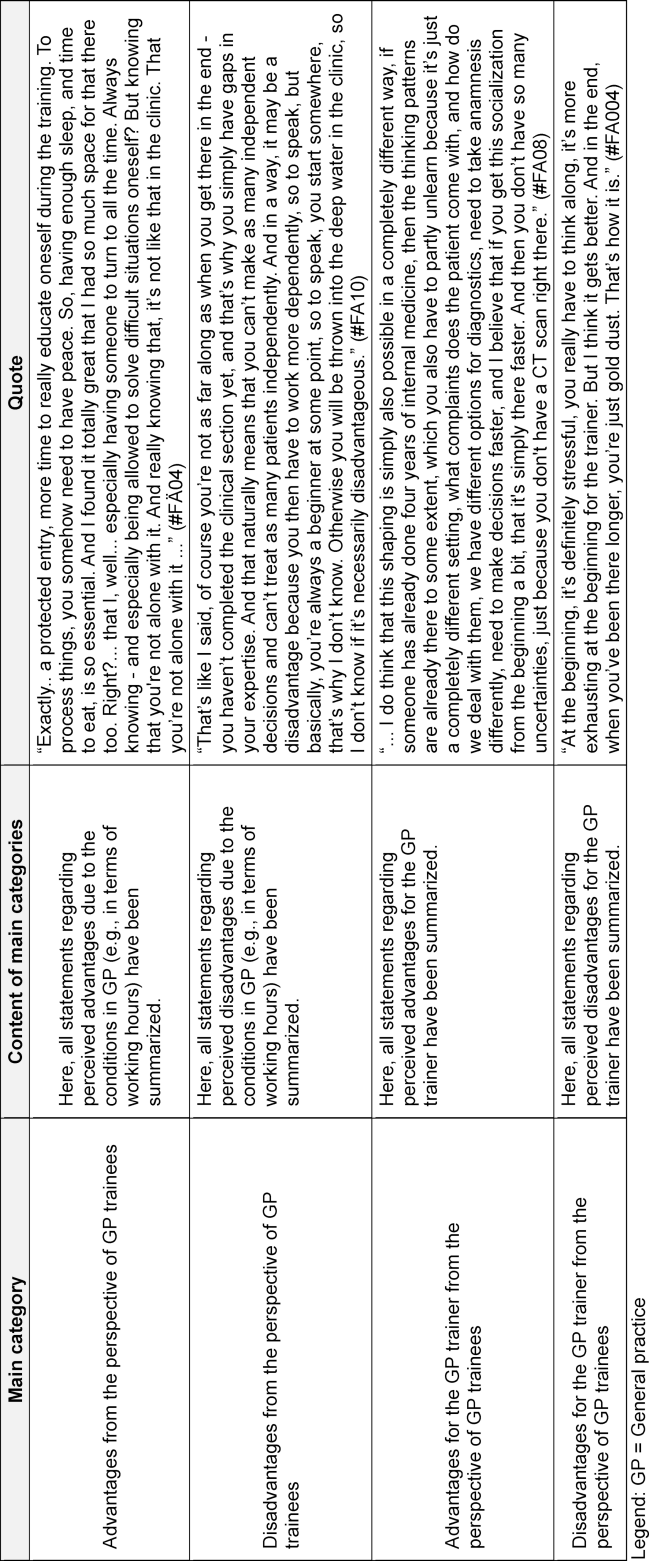
Advantages and Disadvantages of beginning postgraduate training in general practice in the general practitioner’s practice

**Table 4:**
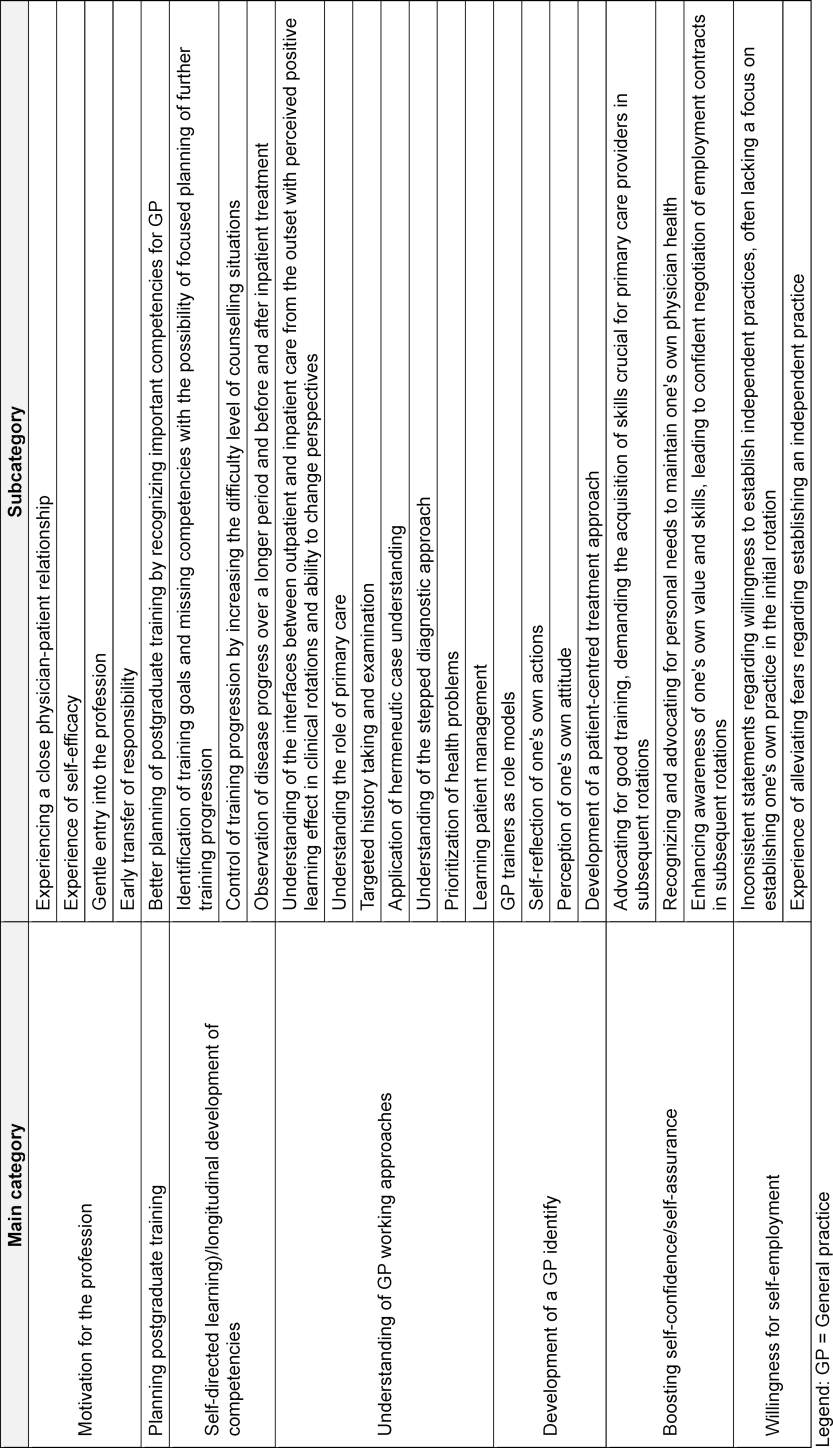
Effects of starting training in general Practice at the general practitioner’s practicee from the perspective of the participating GP trainees
