# Supplementary material for: Starting postgraduate medical training in general practice with a rotation in general practice – a qualitative study on experiences and effects
Source: GMS J Med Educ. 2024 Nov 15;41(5):Doc53. doi: 10.3205/zma001708 (PMC11656185; doi:10.3205/zma001708)
Supplement: Interview guide for general practitioners who have started their training in a general practitioners office [file JME-41-53-s-002.pdf]

Attachment 2: Interview guide for general practitioners who have started their training in a general practitioners office

Attachment 2 to Becker C, Stengel S, Roos M, Altiner A, Schwill S. *Starting postgraduate medical training in general practice with a rotation in general practice – A qualitative study on experiences and effects*. GMS J Med Educ. 2024;41(5):Doc53. DOI: 10.3205/zma001708

To be completed by researchers:	
Nr.	__________________
Datum:	__________________

Starting postgraduate medical training in general practice with a rotation in general practice - A qualitative study on experiences and effects.

Interview guide for general practitioners who have started their training in a general practitioners office

For interviewer – in general

- Excel table number and name – restricted area (N)
- audio file marked by number
- date and time to be checked and documented in recorder
- interviewer has the SMALLEST share of speech
- Be careful – no use of fillers like äh, right, …
- **Open questions**, get people talking, don't give up too early
- Our research objective is to find out the following:
  - impact on the **further course of postgraduate medical training** (with regard to motivation for specialist training in general practice, further rotation planning, longitudinal skills development, the acquisition of medical and general practice competencies) and **later professional activity** (satisfaction with the job description, motivation to set up a medical practice, the desired form of employment or self-employment, the environment of the practice city/state and the preferred form of cooperation).
  - Identification of advantages and disadvantages from the perspective of the GP trainees, but also of the mentors.
  - Identification of challenges/problems caused by the start of postgraduate training in GP care and suitable countermeasures.

Preparation

Introduction

Thank participant for taking part, explain aim of the study and data protection.

Are there further questions?

Before starting the tape capture demographic data

Nr	Sex	Age	Start postgraduate training (year)<br>Weekly working hours<br>Duration first job rotation in GP (months)	Start postgraduate training in which state	Current year of training	Year of specialist examination	Current professional activity (employed/self employed, trainer?; form of establishment; Weekly working hours)	Number of kids

Start

I would start the recording now okay? → ON

1. **name number**
2. **just to make sure, have you given your consent for recording?**
3. **... and you have started your training in the GP sector?**

Good, then here we go..., but really 😊

Part 1 – Effects of starting postgraduate training in GP -> What effects does the start of training in the GP rotation have on further specialist training and on later professional activity?

in brackets () you can find helpful questions for the interviewer

1. What made you decide to go into general practice?

- Have you already completed one third of your practical year in general practice?

2. How did you come up with to start your r training in a GP's office?

- (How did you find your job?; What helped or hindered you in your job search?)

3. How did you experience your rotation?

- (How was the induction phase?; When did you treat patients yourself?; How did you feel?; What uncertainties do you remember?)

- How would you rate your career start compared to colleagues who started their training in the hospital?

4. How did the patients accept you during your first rotation?

- How would you rate this in comparison to later rotations?

5. Were there conflicts with patients due to your status as a job beginner?

- (Can you describe such situations?; How were they resolved?)

6. How did starting your career in a GP’s office influence your further training in general practice?

- How did your first rotation affect the planning of your further rotations? (Note: e.g. ortho due to back pain, paediatrics due to contact with children, ...)

- How did your first rotation affect your personal training goals? (Note: as a supplementary question, if nothing comes up: additional qualifications?)

- Were you able to achieve them?

- How did the rotation influence your motivation to work as a GP? How exactly?

- Did the experience you gained in your first rotation affect your work motivation (GP work or general work motivation) in subsequent rotations? How?

(In other words: Did the first rotation affect your motivation in any way during other rotations? If so, how?)

7. How has the GP rotation affected your skills and attitude? (it's about all the things you need in GP practice; most important skills of a GP)

8. How did your experiences in the first rotation affect your later professional activity or your plans with regard to

- your own practice?

- your preferred working environment (urban/rural)? How? Why?

- The form of employment and/or cooperation you chose (own practice or employed, e.g. as a manager in a MVZ, joint practice, individual practice)? What factors may have influenced your decision during your rotation?

Part 2 - Advantages and disadvantages -> From your own experience, what advantages and disadvantages do specialists in postgraduate training see with regard to starting training in GP care?

9. What advantages would you name regarding the start of further training in GP's office (from the point of view of the GP trainees and from the point of view of the trainers)?

10. What disadvantages would you identify regarding the start of further training in a GP practice? (both from the perspective of the GPs and the trainers)

Part 3 - Tips and conclusions -> What is the best way to deal with any challenges/problems at the start of postgraduate training in a GP's office?

11. What challenges/problems did you experience at the start of further training ina GP's office? (If necessary, refer to answers already given in questions 9 and 10)

- How did you face them?

- How would you face them today in retrospect?

- Was it difficult for you to find a GP trainer? How did you manage to convince him or her?

12. From the perspective of the trainer: looking back, what challenges/problems and what benefits do you see for trainers? (If necessary, refer to answers already given in questions 9 and 10)

13. What advice can you give to young job beginners if they want to start their postgraduate training in a GP's office?

14. Penultimate question: In your opinion, are there any advantages or disadvantages to starting postgraduate training in a GP's office that we have not yet discussed?

15. In summary: in your opinion, what predominates, the advantages or disadvantages and why?

Conclusion: Would you do it again? Yes/No (if no, why not?)

Would you recommend it to others? Yes/No (if no, why?)

As a training supervisor, would you employ job starters in the first year of training? Yes/No (if no, why not?)

Finalisation:

Have we overlooked anything else that is important to you?

OFF – **Thank you for your participation** ☺

Translated with www.DeepL.com/Translator (free version) and www.linguee.de
