# Supplementary material for: Starting postgraduate medical training in general practice with a rotation in general practice – a qualitative study on experiences and effects
Source: GMS J Med Educ. 2024 Nov 15;41(5):Doc53. doi: 10.3205/zma001708 (PMC11656185; doi:10.3205/zma001708)
Supplement: Effects of starting postgraduate training at a general practitioner’s practice – key quotes [file JME-41-53-s-003.pdf]

Attachment 3: Effects of starting postgraduate training at a general practitioner's practice – key quotes

Main category	Content of main category	Quote
Motivation for profession	Statements regarding motivation for general practice and work motivation in subsequent rotations	"That's just my personal story, but it was just fun. It made me happy, it's something I could see myself doing until maybe I retire. And I've seen that it works, that you can balance family and work, that you can do it the way you want, that you have so much freedom and self-efficacy actually. You can really determine everything yourself, and those 2 years, they just absolutely confirmed to me that I want it that way too. And even during that time, I thought to myself, no, in the clinic, I can't imagine working there forever, I can't even decide for myself what I want to do there." (#GP04)
Planning of postgraduate training	Effects on the further course of training	"So, for example, in internal medicine, it was clear to me that I would only do the time I had to. Not much more, and that it wouldn't be my big goal either - I don't know - to become a specialist in nephrology or something like that? So, I could focus quite well. And ultimately, I kind of lived out that focus, in the sense of which areas I got more involved in and which ones I got less involved in. Yeah? So, I could structure myself much better there, while sometimes I have the impression that when you start with internal medicine, you just float around in internal medicine for three years, and do this and that - depending on how it is in the clinic - but you yourself don't really know exactly what you need or want. So, I could proceed a bit more targeted, I think." (#GP008)
Self-*directed learning*/longitudinal development of competencies	Statements on self-directed learning and the opportunity for longitudinal competence development	"... so you really had to remind yourself regularly of this: 'I am a doctor now and I have to take responsibility,' so that you don't get very insecure or scared by the situation, but accordingly, you also quickly grew into it, and that was really/so I was always allowed to ask questions to anyone if I wanted to, but it was also totally tolerated if I worked independently and sometimes looked up a guideline in between, and just decided on my own. And accordingly, I learned to take a high level of personal responsibility after a short time, we also did home visits and all that. There I was allowed to do as much as I wanted and decide everything myself, and I liked that very much. So, I really could actually think about what kind of doctor I wanted to be and treat, and it was conveyed to me that there are many ways to Rome, >laughs< but that I just had to figure out how I wanted to work." (#GP trainee 014)

Attachment 3 to Becker C, Stengel S, Roos M, Altiner A, Schwill S. *Starting postgraduate medical training in general practice with a rotation in general practice – A qualitative study on experiences and effects*. GMS J Med Educ. 2024;41(5):Doc53. DOI: 10.3205/zma001708

Understanding GP working approaches	Statements on the particularities of working in GP practices, the basic principles of general medicine, and the impact of the learned approach on subsequent rotations	"... but somehow, include their perspective (Note: of the patients). Which also in the first rotation >laughs< my GP trainer always emphasized, that you somehow know and take into account the family situation, hobbies, profession, all of that, or simply ask about it... but to be able to establish a secure contact or a relationship of trust in a very short time, and so yes, to look ahead in diagnosis and therapy, right? So, to identify the leading symptom, see what diagnostics have already been done, what are the dangerous courses, maybe plan that well, communicate it well, find a language that the patients understand well, address delicate issues in a friendly manner." (#GP 001)
Development of a GP identity	Summary of statements on identity development, including the development of interests and attitudes through experiences in rotations, through modeling by GP trainers, and through experiencing self-efficacy.	"Yes, I do think that it was such a fundamental building block, this first rotation, that's where you start forming your general practitioner identity for the first time, yes... how is my communication skills, what is my attitude towards certain topics, I think that the first supervisor was also very influential, I do believe that it was very sustainable for me. Because I think, in the last rotation where I was in general practice, my trainer was much less involved with me, I needed less guidance, and so this yes, this attitude, it was actually most reflected upon and discussed in the first segment, and I think that's where I mostly shaped and took it with me." (#GP 008)
Strengthening of self-confidence/self-assurance	Statements about the development and strengthening of self-confidence regarding one's value as general practitioners and professionals.	"For the start, I find it totally important if you learn a bit about it too. You learn how it can be and how it should be, and then I felt that later on, I just didn't want to put up with some things anymore." (#GP 004)
Willingness for self-empolyment	Statements about forming opinions regarding establishing one's own practice.	"Yeah, yeah. Definitely, yeah, it was clear to me then that if I become a general practitioner, I want to establish my own practice, I want to set up on my own, whether it's a team concept or just by myself, I'm not sure yet... more likely in a team, but it definitely showed me that I want to be my own boss." (#GP trainee 03)
		"...it also kind of took away >hesitates< my fear, because I knew before that you could also work as an employee and so on, but when you become a general practitioner, the big goal is always to establish your own practice and so on, I think... the practice also took away my fear of that, or rather the reluctance to take on responsibility and all the work." (#ÄiW018)

Legend: GP = General Practice

Attachment 3 to Becker C, Stengel S, Roos M, Altiner A, Schwill S. *Starting postgraduate medical training in general practice with a rotation in general practice – A qualitative study on experiences and effects*. GMS J Med Educ. 2024;41(5):Doc53. DOI: 10.3205/zma001708
